# Pemphigus vulgaris: a rare clinical image of post-homeopathic necrosis

**DOI:** 10.11604/pamj.2024.48.34.43144

**Published:** 2024-05-30

**Authors:** Leuri Ukey, Jaya Gawai

**Affiliations:** 1 Mental Health Nursing, Smt. Radhikabai Meghe, Memorial College of Nursing, Datta Meghe Institute of Higher education and research, Wardha, India

**Keywords:** Pemphigus vulgaris, autoimmune disorder, life-threatening condition, challenges in diagnosing, diagnosing disease

## Image in medicine

Pemphigus vulgaris is an autoimmune disorder of the skin. It involves blistering and skin and membranes that are mucous. While pemphigus may affect anyone at any age, middle age or older seems to be the most common age for it to appear. Without treatment, some forms of this condition may be fatal and have a prolonged course. It can be more painful and itchy blisters. This blisters in your mouth and then on your skin or genital mucous membranes. A 68-year-old female has been experiencing multiple red-colored lesions with discharge on bilateral left lateral and unilateral. She complained of itching around the border of the lesions and a pins and needles sensation in the middle. she reports multiple ulcers and erosions on her bilateral lower limbs and back. Oral lesions, swelling over legs and face, fluid-filled lesions, and discharge from the lesion. Additionally, she reports multiple ulcers and erosions on her bilateral lower limbs and back, which have worsened in the last 18 days. The patient has also complained of oral lesions for the past eight days. The patient's medical history indicates that she was healthy until six years ago when she started developing small lesions (0.5×0.5cm) on her bilateral legs, which caused burning, itching, and pins and needles sensation. The lesions turned blackish in color and increased in size to approximately 2×1cm after itching. A month later, similar lesions developed on her hands, back, and abdomen, along with blackish discoloration of toes on bilateral feet, which was painful and worsened in the evenings. It will managed through regular follow-up and taking systemic corticosteroids such as Azathioprine, mycophenolate.

**Figure 1:**
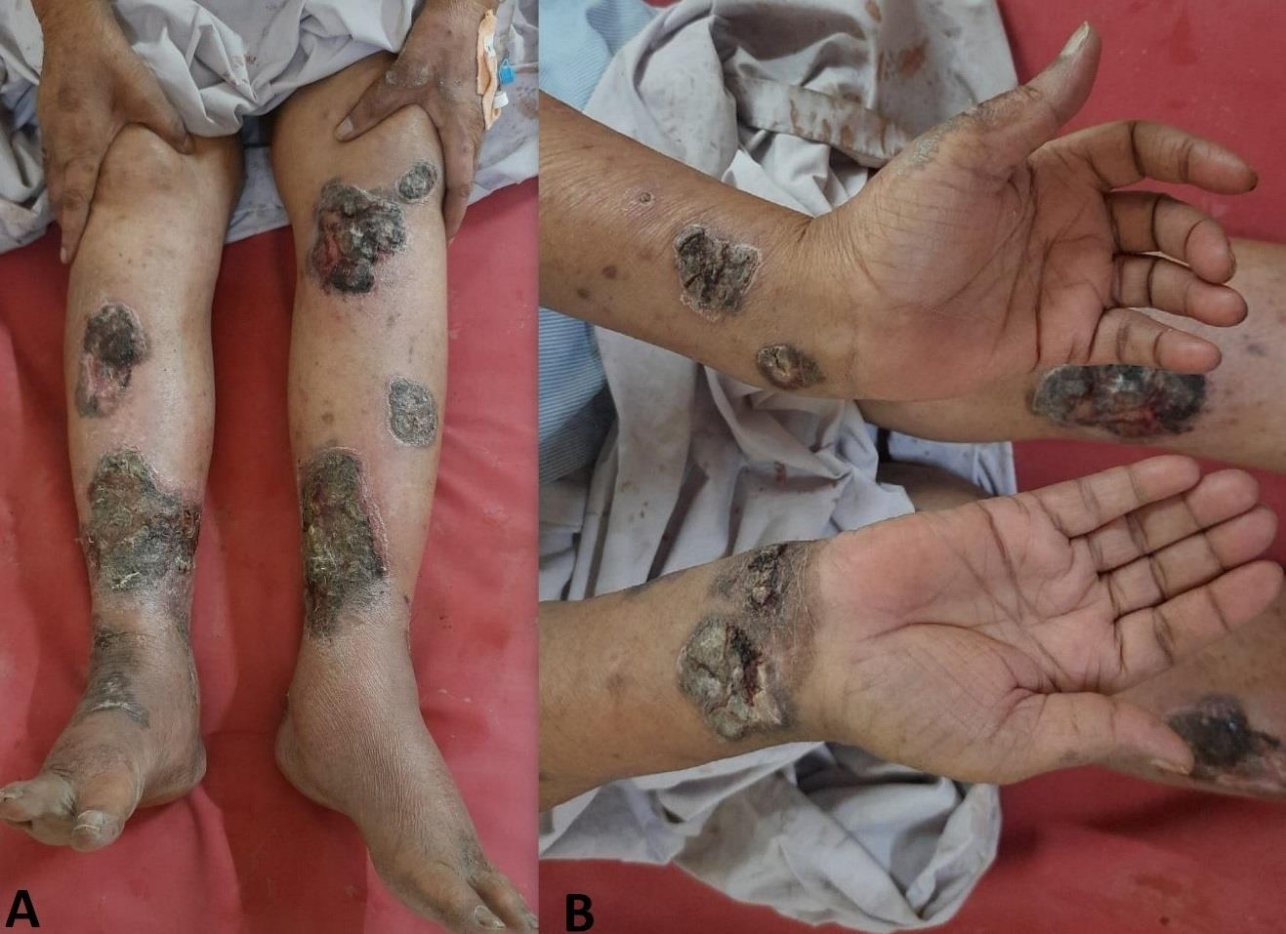
A, B) pemphigus vulgaris, autoimmune disorder, life-threatening condition, challenges in diagnosing, diagnosing disease

